# Opting out against defection leads to stable coexistence with cooperation

**DOI:** 10.1038/srep35902

**Published:** 2016-10-24

**Authors:** Bo-Yu Zhang, Song-Jia Fan, Cong Li, Xiu-Deng Zheng, Jian-Zhang Bao, Ross Cressman, Yi Tao

**Affiliations:** 1 Key Lab of Animal Ecology and Conservation Biology, Chinese Academy of Science, Beijing, China; 2 Laboratory of Mathematics and Complex Systems, Ministry of Education, School of Mathematical Sciences, Beijing Normal University, Beijing, China; 3 Department of Mathematics and Statistics, University of Montreal, Montreal, Canada; 4 School of Complex Systems, Beijing Normal University, Beijing, China; 5 Department of Mathematics, Wilfrid Laurier University, Waterloo, Canada

## Abstract

Cooperation coexisting with defection is a common phenomenon in nature and human society. Previous studies for promoting cooperation based on kin selection, direct and indirect reciprocity, graph selection and group selection have provided conditions that cooperators outcompete defectors. However, a simple mechanism of the long-term stable coexistence of cooperation and defection is still lacking. To reveal the effect of direct reciprocity on the coexistence of cooperation and defection, we conducted a simple experiment based on the Prisoner’s Dilemma (PD) game, where the basic idea behind our experiment is that all players in a PD game should prefer a cooperator as an opponent. Our experimental and theoretical results show clearly that the strategies allowing opting out against defection are able to maintain this stable coexistence.

A great deal of research has been devoted to explain how the evolution of cooperation can be favored by natural selection. Five rules for promoting cooperation based on kin selection^1^, direct and indirect reciprocity^2^,^3^,^4^,^5^, graph selection^6^,^7^ and group selection^8^ have been summarized^9^, and these models provided simple conditions that natural selection can lead to full cooperation. However, few literatures have considered how cooperation and defection can coexist in the long-term even though this phenomenon is common in nature and human society^10^. Other studies^11^,^12^ have shown ongoing oscillations between cooperative and defective societies can evolve in theoretical models, possibly explaining such phenomena as the alternate appearance of war and peace^11^. However, these models still do not provide a simple mechanism to drive the long-term stable coexistence of cooperation and defection.

Cooperation means that a donor pays a cost, *c*, for a recipient to get a benefit, *b*, where *b* > *c*^11^,^12^. In the corresponding one-shot PD game, defection is the only Nash equilibrium (NE)^11^,^12^. On the other hand, for the repeated PD game with two strategies TFT (tit-for-tat) and AllD (always defect), TFT is a NE if the expected number of iterated interactions between a pair of individuals is larger than the critical value *b*/(*b *− *c*)^3^,^4^,^9^,^11^,^12^. However, the stable coexistence of TFT and AllD is impossible in the TFT-AllD game. Clearly, the success of TFT is mainly due to the increased chance of interactions between cooperators^4^,^13^. That is, TFT provides a mechanism whereby cooperators preferentially interact among themselves. Similarly, assortative matching among cooperators has been used to explain why altruism can emerge^14^,^15^,^16^,^17^,^18^, although the evolutionary origin of the non-uniform interaction rates among cooperators has not been explained^17^,^18^.

For the repeated PD game, one of the key assumptions is that the interaction between a pair of individuals will be repeated for several rounds, and no player in the game is able to stop the interaction with his/her opponent^4^,^11^,^12^,^13^. However, based on individual self-interest in the PD game, both cooperators and defectors prefer an opponent who cooperates (i.e. only cooperator is always welcome). Thus, if players are able to unilaterally terminate the interactions with their opponents, then a simple rule will be followed by all individuals: I would like to keep my opponent if he/she is a cooperator; and if my opponent is a defector, I will stop the interaction with him/her and seek a new partner instead.

Recently, an interesting study based on the concept of conditional dissociation found that a strategy called “out-for-tat” (OFT) is important for the coexistence of cooperation and defection^19^,^20^,^21^,^22^,^23^,^24^,^25^,^26^. Since OFT means that an individual displaying cooperation (C) will respond to defection (D) by merely leaving, OFT will not tolerate defection but, unlike TFT, it does not seek revenge. To reveal the fundamental evolutionary force driving the coexistence of C and D, we conduct a simple experiment based on the repeated PD game, where, unlike the classic repeated game, each player can unilaterally break off the pairwise interaction with his/her opponent according to his/her own volition. On the other hand, different from previous experiments on repeated PD game with outside option^19^,^20^,^21^,^22^,^23^,^24^,^25^,^26^, the expected number of rounds between a pair of individuals is still limited in our experimental design even if these two individuals would like to continue their interaction^4^,^11^,^12^,^13^.

## Results

A total of 264 university students were divided into five groups, including two control groups (C1 and C2) and three treatment groups (T1, T2 and T3) (Supplementary Information (SI), Section 1.1). Note that the experimental settings in all three treatment groups T1, T2 and T3 are exactly the same, therefore in the data analysis we treat them as one group, denoted by T (SI, Section 1.2). The basic payoff matrix in our experiment is


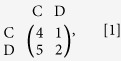


where this payoff matrix can be normalized as a simplified PD game with *b* = 3 and *c* = 1. Each subject participated in 65 to 80 rounds of interactions between pairs of individuals playing this game over about 40 minutes. Participants were told that the experiment would be randomly stopped at 60–80 rounds. Thus, to avoid end-round effects and to keep the comparison unbiased, we only used data in the first 60 rounds in all groups in later statistical analysis.

The control experiments C1 and C2 are the classic repeated PD game and one-shot PD game, respectively. In C1, each interaction pair continues to the next round with probability 5/6 and is terminated with probability 1/6. At the end of each round, all single subjects form new interaction pairs through random meeting in the next round. In C2, all subjects are shuffled to form new interaction pairs in every round. On the other hand, the experimental setting in the treatment T is similar to C1 except that, at the end of each round, each subject decides whether he/she would like to continue the interaction in the next round with his/her current opponent. An interaction pair is terminated if at least one of the two subjects decides to stop; and is automatically terminated by the system with probability 1/6 even if both subjects choose to continue. After that, similarly, all single subjects are randomly repaired with a new opponent to play in the next round (SI, Section 1.1).

**Figure 1:**
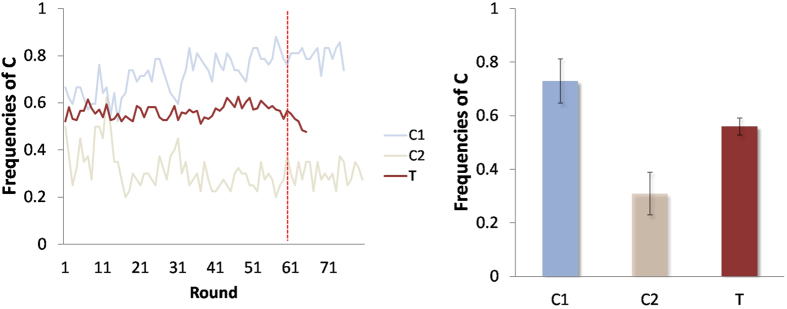
Cooperation levels per round for treatment compared to control experiments. Panel (**a**) shows the time evolution of cooperation levels per round in C1, C2 and T respectively, with dashed line at round 60. Panel (**b**) shows the average cooperation levels over 60 rounds with standard errors in C1, C2 and T, respectively, which are: 0.72 ± 0.0808 in C1; 0.32 ± 0.0876 in C2; and 0.56 ± 0.0287 in T. Mann-Whitney U-test shows that the differences between C1 and C2, between C1 and T and between T and C2 are significant with *p*-value < 0.01 (after Bonferroni correction) (SI, Table S3).

The primary experimental result (Fig. 1) is that for all control and treatment experiments, the cooperation level (defined as the frequency that C is used) in C1 is significantly higher than either in treatment T or in control C2, and that in T is significantly higher than in C2. (SI, Section 1.2).

It is also easy to see that the cooperation level in C1 increased over time from an average of 64% in the initial 10 rounds to an average of 80% from round 51 to 60. This time evolution of C in C1 can be characterized well by the TFT-AllD game, where both TFT and AllD are evolutionarily stable with current parameters^11^,^12^. In particular, the system state tends to TFT given the initial data because it has a larger basin of attraction under the evolutionary dynamics. In C2, the cooperation level is much lower, declining from 39% in the initial 10 rounds to 28% from round 51 to 60. Obviously, C2 reflects well the characteristics of the one-shot PD game where only D is evolutionarily stable. However, in T, a relatively stable cooperation level is maintained over all rounds (e.g. an average of 56% in the initial 10 rounds and 58% from round 51 to 60). Moreover, the cooperation level in T is between the cooperation levels in C1 and in C2. This suggests that the treatment T provides a possible mechanism to maintain the stable coexistence of C and D.

**Figure 2:**
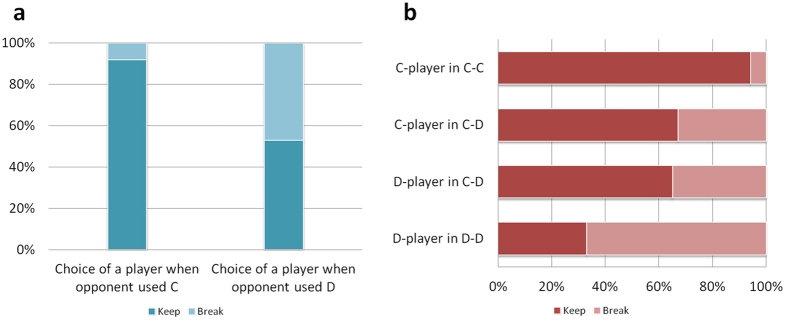
Individuals’ responses to the behavior of their opponents in the first 60 rounds. Panel (**a**) shows the probability that, at the end of each round, a player chooses to keep, or break, the interaction with his/her opponent who uses C (D). Panel (**b**) shows the probabilities that, at the end of each round, a player using C (D) chooses to keep, or break, the interaction when his/her opponent uses C and when his/her opponent uses D.

In the treatment T, the chance that a subject decides to continue the interaction with his/her current opponent in the next round is 92% if his/her opponent displays C, whereas this chance drops to 53% if his/her opponent displays D (Fig. 2). We are also interested in how a player using strategy A responds when his/her opponent displays strategy B, where A, B = C, D. For the interaction pair C-C, only 10% of the interactions are stopped by the players, with 94% of the C-players choosing to continue the interaction with their current opponent; for the interaction pair C-D, the probability that at least one player chooses to stop the interaction is 56%, in which C-players (respectively, D-players) choose to stop the interaction with probability 33% (respectively 35%); and for the interaction pair D-D, 67% of the interactions are terminated by the players, in which each D-player chooses to stop the interaction with probability 43% (Fig. 2).

We then identify the subjects who stop the interactions with significantly higher probability when their opponents display D than that when their opponents display C using two sided binomial sample test with 95% confidence intervals. These subjects are called OFT-strategists. According to this standard, most of the subjects (85.16%) can be classified as OFT-strategists. Only 3.3% of the subjects stop their interactions with significantly higher probability when their opponents display C than when they display D. The remaining 11.54% of the subjects cannot be identified (i.e., the chance that they will stop the interactions when their opponents display D is not significantly different from the chance when their opponents display C).

## Discussion

**Figure 3:**
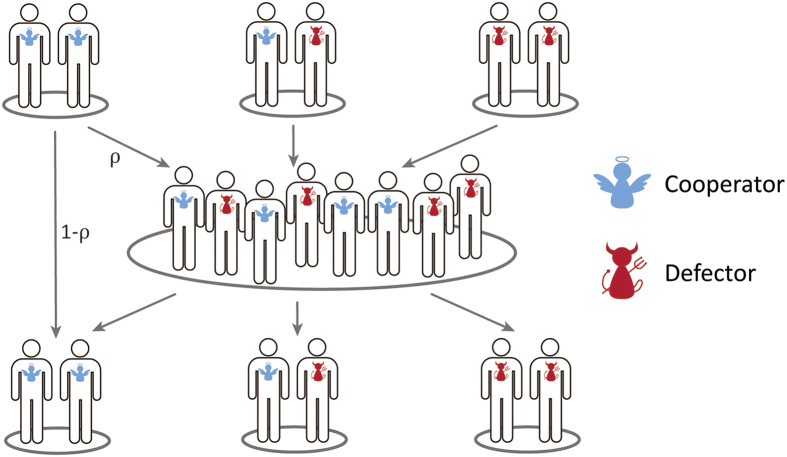
The setup of the evolutionary model. OFT-cooperators and OFT-defectors are marked by blue angels and red fiends, respectively. At the end of a round, C-D pairs and D-D pairs will be broken since all individuals immediately stop the interaction with a defector, and a C-C pair will be terminated with probability *ρ* even though both individuals are willing to continue. These single individuals will be paired with a new partner through random meeting in the next round.

To reveal the mechanism behind the treatment T maintaining the coexistence of C and D, we develop a concise theoretical framework to show how opting out against D leads to the coexistence of C in PD game settings. Consider a simplified repeated PD game^11^,^12^ with payoff matrix 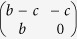 (analysis for the general PD game is shown in SI, Section 2.2). At the end of each round, each player can unilaterally break off the interaction with his/her opponent according to his/her own volition. In fact, we assume that all individuals (including both cooperators and defectors) respond to D by merely leaving (i.e., using OFT)^25^,^26^. Moreover, as in the classic repeated game, the interaction between a pair of individuals is terminated after each round with probability *ρ* even if these two individuals would like to continue their interaction^4^,^11^,^12^,^13^. Thus, the probability that an interaction pair C-C continues in the next round is 1−*ρ*, implying that the expected length of their interaction is 1/*ρ*. On the other hand, the interaction pairs C-D and D-D will never continue to the next round, becoming single individuals immediately. At the end of each round, all single individuals form new interaction pairs through random meeting in the next round (Fig. 3).

**Figure 4:**
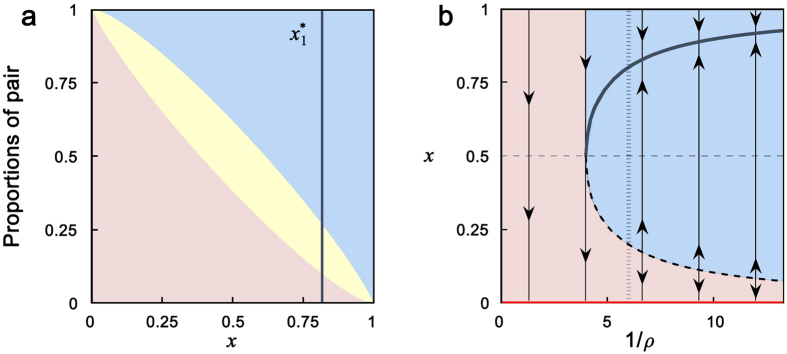
Evolutionary dynamics of Eq. [2] with *b* = 3 and *c* = 1. (**a**) Blue, yellow and pink regions represent respectively the proportions of C-C, C-D and D-D pairs for all possible 0 < *x* < 1 at the temporal equilibrium with 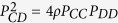 (Eq. [S3] in SI, Section 2.1), where the parameter *ρ* is taken as *ρ* = 1/6; and the blue line denotes the stable interior equilibrium 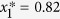. (**b**) Phase portrait of the dynamics Eq. [2] for different *ρ*. The red line denotes the stable boundary *x* = 0, the solid blue curve denotes the stable interior equilibrium 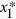 (which is bigger than 1/2), and the dashed curve denotes the unstable interior equilibrium 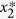. The population evolves to the boundary *x* = 0 for initial *x* in the pink region, and the dynamics leads to a stable coexistence of C and D for initial *x* in the blue region. The inverse 1/*ρ* represents the expected number of interactions of a C-C pair, where the vertical dash line denotes 1/*ρ* = *6*.

Based on the theoretical analysis in SI, Section 2.1, the time evolution of the frequency of OFT-cooperators, denoted by *x*, can be modeled by the replicator dynamics^27^,^28^


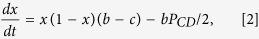


where the frequency *P*_*CD*_ of C-D pairs is shown to be given by Eq. [S3] in SI, Section 2.1. The stability analysis of this dynamics shows that (i) the boundary *x* = 0 is locally asymptotically stable for all possible 0 < *ρ* < 1 but the boundary *x* = 1 is never stable; (ii) two interior equilibria (Eq. [S5] in SI, Section 2.1), denoted by 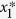 and 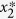 with 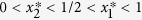, exist if *ρ* < (*b* − *c*)^2^/(*b* + *c*)^2^, and 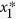 is locally asymptotically stable and 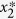 is unstable; (iii) a unique unstable interior equilibrium x^*^ = 1/2 exists if *ρ* = (*b* − *c*)^2^/(*b* + *c*)^2^; and (iv) the boundary *x* = 0 is globally asymptotically stable if *ρ* > (*b* − *c*)^2^/(*b* + *c*)^2^ (Fig. 4). Similar results for the general PD game are obtained in SI, Section 2.2.

Clearly, there are some differences between this simple theoretical model and the experimental data in the treatments. In particular, the theoretical model assumes that all C-D pairs and D-D pairs will be terminated by the players, whereas in the experiments, the termination rate of such interaction pairs was 72% and subjects sometimes used D to response D (i.e., they adopted TFT-like strategies, see SI Section 1.3 for details). Since more interactions with defectors were continued to the next round, it is therefore not surprising that the observed frequency of cooperation of 0.56 is less than the theoretically predicted stable equilibrium level 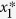 of 0.82 for our parameters (Fig. 4(a)). Nevertheless, the experiment and theory both show that adding the option of opting out can lead to the stable coexistence of C and D.

In conclusion, our experimental results and theoretical analysis that emerge from allowing individuals to opt out against defection show that this elementary mechanism based on direct reciprocity promotes the stable coexistence of cooperation and defection. These outcomes are especially important since stable coexistence is such a commonly observed phenomenon^1^ that does not occur for models of the repeated PD game that typically analyze such strategies as AllD and AllC in combination with others based on direct reciprocity (e.g. TFT, generous-TFT and win-stay lose-shift)^11^,^12^. This supports our contention that, while we agree with the generally recognized opinion that direct reciprocity is the most important force driving the evolution of cooperation^4^,^29^, strategies allowing opting out provide a better general framework for its analysis.

We now discuss some aspects of the experimental design and theoretical models, and review related literature.

In the experimental design, we assumed that the expected number of rounds in an interaction was limited even when there was an outside option because of the following two reasons. First, in the real world, an interaction may be terminated due to unexpected reasons even if both individuals would like to continue. In addition, this assumption allows us to compare the experimental results in T and C1 directly.

In the theoretical model, we assumed all individuals would respond to defection by leaving. However, many subjects in experiments used D as a response to D. A possible explanation of these behaviors would be direct reciprocity, e.g., these subjects want to punish their opponents by defection. Furthermore, there are also some subjects responded to D by C. They may expect that their kindness can encourage their opponent to cooperate in the future. Whatever the ultimate reasons behind these non-OFT behaviors are, it is also important to verify theoretically whether individuals not using OFT can successfully invade a population consisting of OFT-cooperators and OFT-defectors. It is easy to see that the expected payoff of an individual displaying *C* (or *D*) but not using OFT can be no higher than that of an OFT- cooperator (or OFT- defector) since the chance that an individual not using OFT is paired with an opponent displaying *C* will be less than that of an individual using OFT. Thus, when all individuals use OFT and the system state is at an interior stable equilibrium 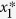, an individual not using OFT cannot successfully invade this population since OFT- cooperators and OFT- defectors have the same expected payoff at this equilibrium.

We note that there exist three classes of literature investigating the effect of outside option on cooperation, but their focus and results are different from ours. One class considers infinitely repeated PD game, where an interaction is terminated only if one of individuals in the partnership chooses to stop^19^,^20^,^21^,^22^,^23^,^24^,^30^, or is deemed dead by the system^25^,^26^. These studies often focus on Nash equilibrium (NE) or a certain class of strategies. For instance, it has been shown the game has no pure strategy NE because trust building strategies can defeat defectors^24^, and that TFT is dominated by some conditional and unconditional strategies^25^,^26^. Another class allows abstaining from a game^21^,^22^,^31^,^32^, with players choosing between an outside option (i.e., to be a loner) and the PD game. In such case, cooperators, defectors and loners can coexist if the payoff of the outside option is higher than the payoff of mutual defection. However, voluntary participation usually does not lead to a stable equilibrium, but to an unending limit cycle^31^,^32^. The third class is developed on graph selection, arguing that dynamical networks where subjects can update their network connections can lead to cooperative outcomes^33^,^34^,^35^,^36^,^37^,^38^. When subjects play several PD games simultaneously with their neighbors, they often preferentially break social links with defectors and form new links with cooperators, which creates an incentive to cooperate^35^,^36^,^37^,^38^. Our model can be seen as the simplest dynamical networked PD game, where each individual only connects to one partner. However, network reciprocity, such as cooperators have more connections on average than defectors^36^,^37^, or cooperators form large cooperative clusters^35^,^38^, are not included in our model.

Although the three classes of models contain the idea of walking away from the interaction with defectors, it cannot be simply concluded that outside option promotes the coexistence of cooperation and defection. Because other assumptions in these models, such as infinitely repeated game^19^,^20^,^21^,^22^,^23^,^24^,^30^, optional participation^31^,^32^ and spatial reciprocity^33^,^34^,^35^,^36^,^37^,^38^, may have positive effects on cooperation. Thus, our theoretical analysis and experimental results provide convincing evidences that opting out against defection alone is enough to maintain the stable coexistence of cooperation and defection.

There are also existing studies^11^,^12^ that discuss the long-term evolutionary dynamics of cooperation and defection based on the repeated PD game. They have shown evolutionary cycles of cooperation and defection (i.e. from AllD to TFT to GTFT to AllC and back to AllD) can exist, and suggest that societal oscillation between cooperation and defection is a fundamental part of all our observations regarding the evolution of cooperation. However, compared to these studies, our model provides a very simple mechanism such that stable coexistence of cooperation and defection is possible without oscillation.

## Methods

### Experimental design

The experiments were conducted in computer labs at Beijing Normal University on April 2th and April 3th, 2015. The treatment group T1 was conducted on April 2th, and groups T2, T3, C1 and C2 were conducted on April 3th. All 264 participants were undergraduate students from Beijing Normal University who had no background in game theory and economics. The interactions between participants were anonymous, and via the computers. In the experiments, the participants were separated by the frosted-glass such that they could not see each other’s computer screen, and they were not allowed to communicate during the experiment (SI, Figure S1).

Before the experiment started, the rules of the game were explained to all participants, who were also shown the instructions of the experiment (in Chinese) for their particular control or treatment group. To ensure that all participants fully understand the game, they were required to answer correctly 4–5 questions before logging in to the formal experiments. The total number of rounds (or length) of the formal experiment for each of groups was about 65–80 (taking about 40 minutes), and the participants were told that the experiment would be randomly stopped at 60–80 rounds. Although there is no time limitation for participants’ decision making in each round, it was recommended that participants submit their decisions within 30 seconds (there was a 30 second countdown on the screen).

When the experiment for each of groups was finished, the score of each participant in the experiment was converted to Chinese Yuan (CNY) with ratio 1: 0.3. The payoff of each participant plus a fixed amount of 20 Yuan was his/her total earning in the experiment. The overall average earning in our experiments was 83.9 Yuan (with minimum 63 Yuan, and maximum 108 Yuan); the group average earning is 80.6 Yuan (with minimum 63 Yuan, and maximum 95 Yuan) in T1, 79.3 Yuan (with minimum 66 Yuan, and maximum 91 Yuan) in T2, 83 Yuan (with minimum 72 Yuan, and maximum 96 Yuan) in T3, 98 Yuan (with minimum 83 Yuan, and maximum 108 Yuan) in C1, and 83 Yuan (with minimum 66 Yuan, and maximum 96 Yuan) in C2.

### Ethics

All participants provided written informed consent after the nature and possible consequences of the studies were explained. All experimental methods were carried out in accordance with the approved guidelines. All experimental protocols were approved by the Ethics Review Committee of Institute of Zoology.

## Additional Information

**How to cite this article**: Zhang, B.-Y. *et al*. Opting out against defection leads to stable coexistence with cooperation. *Sci. Rep.* **6**, 35902; doi: 10.1038/srep35902 (2016).

## Supplementary Material

Supplementary Information
