# Supplementary material for: Opting out against defection leads to stable coexistence with cooperation
Source: Sci Rep. 2016 Oct 24;6:35902. doi: 10.1038/srep35902 (PMC5075917; doi:10.1038/srep35902)
Supplement: Supplementary Information [file srep35902-s1.pdf]

Opting out against defection leads to stable coexistence with cooperation

Bo-Yu Zhang[1,2,†], Song-Jia Fan[1,†], Cong Li[1,3,†], Xiu-Deng Zheng[1,†], Jian-Zhang Bao[4], Ross Cressman[5,*] and Yi Tao[1,*]

[1]Key Lab of Animal Ecology and Conservation Biology, Chinese Academy of Science, Beijing, China

[2] Laboratory of Mathematics and Complex Systems, Ministry of Education, School of Mathematical Sciences, Beijing Normal University, Beijing, China

[3]Department of Mathematics and Statistics, University of Montreal, Montreal, Canada

[4]School of Complex Systems, Beijing Normal University, Beijing, China

[5]Department of Mathematics, Wilfrid Laurier University, Waterloo, Canada

†These authors contributed equally to this work.

[*] Authors for correspondence: Yi Tao (yitao@ioz.ac.cn) and Ross Cressman (rcressman@wlu.ca)

Supplementary information

1. Experimental Design and Results

1.1. Experimental design and description

The experiments based on the Prisoner's Dilemma (PD) game were conducted in 5 groups, of which 2 are control groups, denoted by C1 and C2 respectively, and 3 are treatment groups denoted by T1, T2 and T3 (note that the experimental settings in all three treatment groups T1, T2 and T3 are exactly the same). The payoff matrix of the PD game is taken as

$$\begin{array}{cc} & \begin{array}{cc} \mathrm{C} & \mathrm{D} \end{array} \\ \begin{array}{c} \mathrm{C} \\ \mathrm{D} \end{array} & \begin{pmatrix} 4 & 1 \\ 5 & 2 \end{pmatrix} \end{array},$$

where C denotes the cooperation and D the defection. This payoff matrix can be normalized as

$$\begin{pmatrix} 4 & 1 \\ 5 & 2 \end{pmatrix} = \begin{pmatrix} 2 & 2 \\ 2 & 2 \end{pmatrix} + \begin{pmatrix} 2 & -1 \\ 3 & 0 \end{pmatrix} = \begin{pmatrix} 2 & 2 \\ 2 & 2 \end{pmatrix} + \begin{pmatrix} b-c & -c \\ b & 0 \end{pmatrix},$$

where $b=3$ and $c=1$. This simplified PD game has benefit to cost ratio $b/c=3$.

Each subject in an interaction pair chooses C or D in each round. At the end of each round, the players' choices and payoffs are shown to both of them (on the computer screen). For different groups, the experimental designs are given below.

(1) C1 group follows the classic repeated PD games, in which each interaction pair is automatically stopped by the system at the end of each round with probability $\rho=1/6$, and is continued in the next round with complementary probability $1-\rho=5/6$. If an interaction pair is automatically terminated, then both players are randomly re-paired with new opponents in the next round (Table S1). In the experiment, each round has at least 4 single subjects (note that each round has on average 7 single subjects), so we can guarantee that players will not meet their previous partner in the next round.

(2) C2 group follows the classic one-shot PD game, in which each interaction pair is terminated at the end of each round with probability $\rho = 1$. All players are then randomly re-paired in the next round (Table S1).

(3) Three treatment groups T1, T2 and T3 also follow the repeated PD game (similar to C1 group), but a new option is added for each player whereby he/she unilaterally stops the interaction with his/her opponent at the end of each round even if his/her opponent prefers to continue the game in next round. Thus, in three treatment groups (T1, T2 and T3), there are two reasons an interaction pair may stop at the end of each round. One is that the interaction is automatically stopped by the system with probability $\rho = 1/6$, and the other is that at least one player in the interaction pair unilaterally stops the interaction with his/her opponent. At the end of each round, not only the choice and payoff of each player and his/her opponent's choice and payoff are shown on the computer screen but also the reason why the interaction stops is shown when this occurs. If an interaction pair is stopped, then both players are randomly re-paired with new opponents in the next round (Table S1). In the experiment, each round has at least 4 single subjects, so we can guarantee that players will not meet their previous partner in the next round.

Table S1. Experimental design

	T1	T2	T3	C1	C2
Group size	60	66	56	42	40
Parameters	$b=3, c=1, \rho=1/6$			$b=3, c=1, \rho=1/6$	$b=3, c=1, \rho=1$
Option: Opting out	Yes			No	No
Number of rounds	65	65	65	75	80

1.2. Basic data analysis

The proportion of cooperation (C), denoted by P_C, in each round for each of C1, C2 and T (where T is the collection of T1, T2 and T3 since T1, T2 and T3 have the same experimental design) is shown in Figure S1a (i.e. time evolution of C) (see also Figure 1 in the main text), where the average of P_C in the first 60 rounds is 0.72 in C1, 0.32 in C2 and 0.56 in T (Table S2, Figure S2).For the average of P_C, C1 is significantly larger than T, and T is significantly larger than C2 (Table S3). From Figure S1a, it is clear that the cooperation level (P_C) slowly increases from 0.64 to 0.8 in C1, slowly decreases from 0.39 to 0.28 in C2, and maintains a constant level of about 0.56 in T. Furthermore, to show the cooperation level in each of T1, T2 and T3, the proportion of C per round for each of T1, T2 and T3 is also shown in Figure S3a, where the average of P_C in the first 60 rounds is 0.56 in T1, 0.52 in T2 and 0.62 in T3 (Table S2), and the differences between T1 and T2, between T1 and T3, and between T2 and T3 are not significant (Table S3, Figure S2).

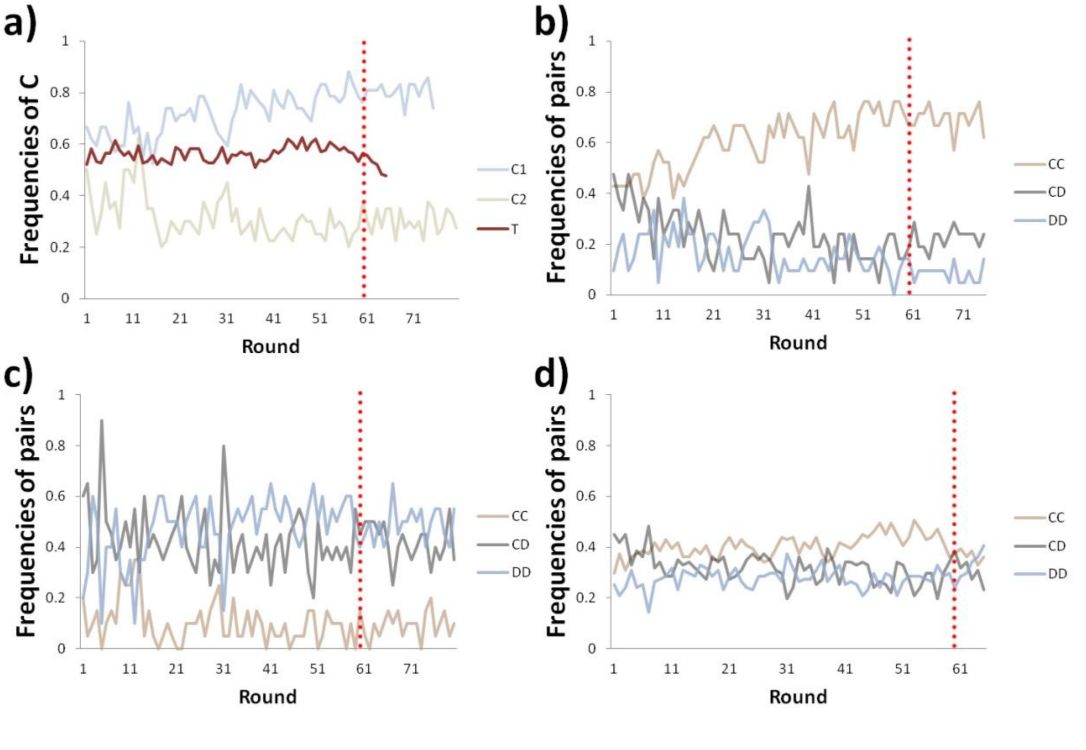

Figure S1. Panel (a) shows P_C per round for each of C1, C2 and T. Panels (b), (c) and

(d) show P_{CC}, P_{CD} and P_{DD} per round in C1, C2 and T, respectively. The dotted lines mark at round 60.

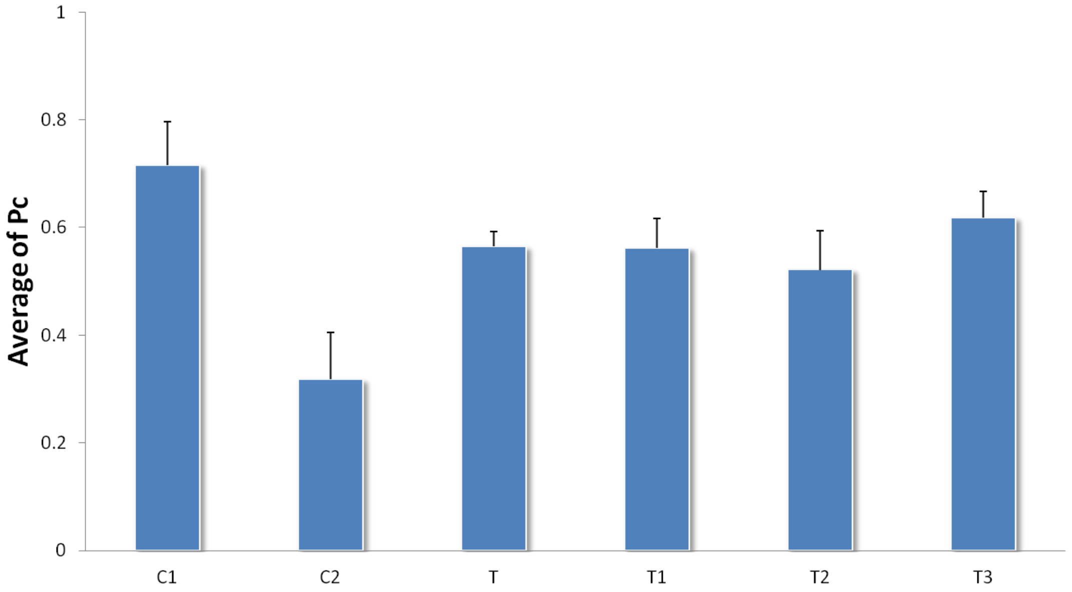

Figure S2. Average P_C in C1, C2 and T (T1, T2, T3) with standard errors in the first 60 rounds, which is 0.72 in C1, 0.32 in C2, 0.56 in T (0.56 in T1, 0.52 in T2, 0.62in T3).

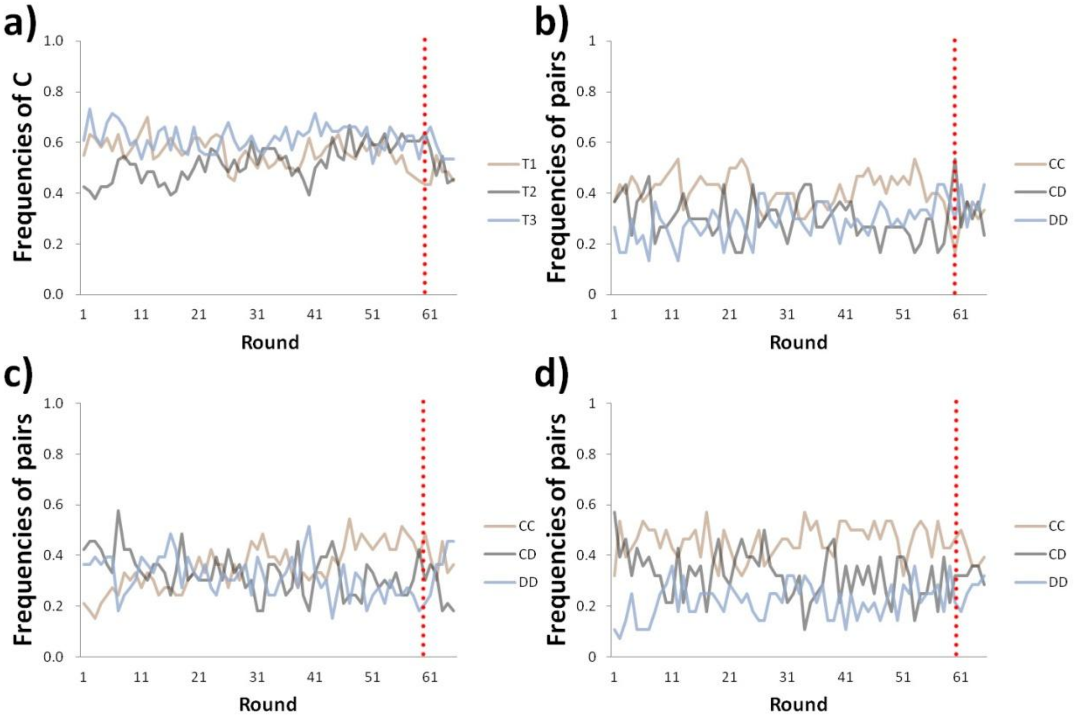

Figure S3. Panel (a) shows P_C per round for each of T1, T2 and T3. Panels (b), (c)

and (d) show P_{CC}, P_{CD} and P_{DD} per round in T1, T2 and T3, respectively. The dotted lines mark at round 60.

Table S2. Average of P_C, and averages of P_{CC}, P_{CD} and P_{DD} for each of C1, C2, T (T1, T2, T3)

Average	C1	C2	T	T1	T2	T3
P_C	0.72	0.32	0.56	0.56	0.52	0.62
P_{CC}	0.60	0.10	0.40	0.41	0.35	0.46
P_{CD}	0.23	0.43	0.32	0.30	0.33	0.32
P_{DD}	0.17	0.47	0.28	0.29	0.31	0.22

Table S3. Mann-Whitney U-test for difference in the average of P_C ($n_1 = 60$, $n_2 = 60$) between C1 and C2, between C1 and T, between C2 and T, between T1 and T2, between T1 and T3, and between T2 and T3, where the symbol "*" denotes that the difference is significant at $\alpha = 0.01/12 = 8.3\text{E-}4$ (with Bonferroni correction).

	C1	C2	T1	T2	T3
C1	1	< 1E-6*	< 1E-6*	< 1E-6*	< 1E-6*
C2	< 1E-6*	1	< 1E-6*	< 1E-6*	< 1E-6*
T	< 1E-6*	< 1E-6*			
T1	< 1E-6*	< 1E-6*	1	0.002	< 1E-6*
T2	< 1E-6*	< 1E-6*	0.002	1	< 1E-6*
T3	< 1E-6*	< 1E-6*	< 1E-6*	< 1E-6*	1

Notice that, for each interaction pair, there are three possible strategy-pairs in each round, which are C-C, C-D and D-D, respectively (for example, if one player displays C and his/her opponent also displays C, then this strategy-pair is denoted by C-C). For convenience, the proportions of interaction pairs C-C, C-D and D-D in each

round are denoted by P_{CC}, P_{CD} and P_{DD}, respectively. Similar to the analysis of P_C, the proportions of interaction pairs C-C, C-D and D-D (i.e. P_{CC}, P_{CD} and P_{DD}) per round for each of C1, C2 and T are plotted in Figure S1b, in Figure S1c and in Figure S1d, respectively. The averages of P_{CC}, P_{CD} and P_{DD} in the first 60 rounds are 0.60, 0.23 and 0.17 in C1; 0.10, 0.43 and 0.47 in C2; and 0.40, 0.32 and 0.28 in T (Table S2, Figure S4). For the averages of P_{CC}, P_{CD} and P_{DD} in each of C1, C2 and T, the difference between C1 and T and the difference between C2 and T are all significant (Table S4). Furthermore, for each of T1, T2 and T3, the proportions P_{CC}, P_{CD} and P_{DD} per round are plotted in Figure S3b, Figure S3c and Figure S3d, respectively. The averages of P_{CC}, P_{CD} and P_{DD} are 0.41, 0.30 and 0.29 in T1; 0.35, 0.33 and 0.31 in T2; and 0.46, 0.32 and 0.22 in T3 (Table S2, Figure S4,Table S4).

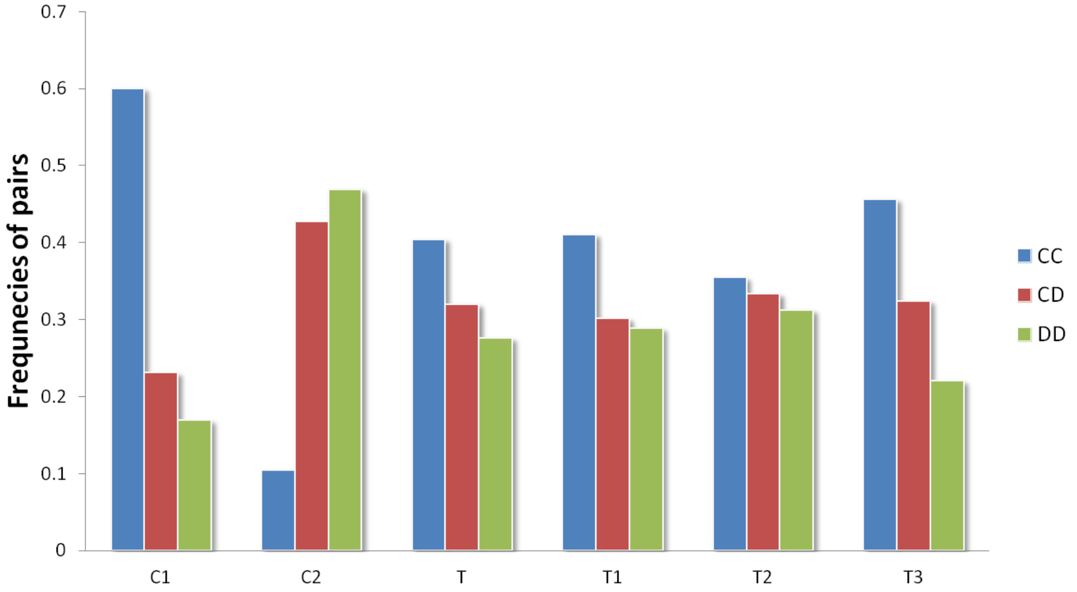

Figure S4. Averages of P_{CC}, P_{CD} and P_{DD} in C1, C2 and T (T1, T2, T3) in the first 60 rounds, which are 0.60, 0.23, 0.17 in C1; 0.10, 0.43, 0.47 in C2; 0.40, 0.32, 0.28 in T (0.41, 0.30, 0.29 in T1; 0.35, 0.33, 0.31 in T2; 0.46, 0.32, 0.22 in T3).

Table S4. Mann-Whitney U-test for difference in the averages of P_{CC}, P_{CD} and

P_{DD} ($n_1 = 60$, $n_2 = 60$) between C1 and C2, between C1 and T, between C2 and T, between T1 and T2, between T1 and T3, and between T2 and T3, where the symbol "*" denotes that the difference is significant at $\alpha = 0.01/12 = 8.3\text{E-}4$ (with Bonferroni correction).

(a) Mann-Whitney U-test for difference in the average of P_{CC}

	C1	C2	T1	T2	T3
C1	1	< 1E-6*	< 1E-6*	< 1E-6*	< 1E-6*
C2	< 1E-6*	1	< 1E-6*	< 1E-6*	< 1E-6*
T	< 1E-6*	< 1E-6*			
T1	< 1E-6*	< 1E-6*	1	9.6E-5*	0.004
T2	< 1E-6*	< 1E-6*	9.6E-5*	1	< 1E-6*
T3	< 1E-6*	< 1E-6*	0.004	< 1E-6*	1

(b) Mann-Whitney U-test for difference in the average of P_{CD}

	C1	C2	T1	T2	T3
C1	1	< 1E-6*	3.5E-5*	< 1E-6*	1E-6*
C2	< 1E-6*	1	< 1E-6*	2.5E-5*	6E-6*
T	< 1E-6*	< 1E-6*			
T1	3.5E-5*	< 1E-6*	1	0.015	0.199
T2	< 1E-6*	2.5E-5*	0.015	1	0.614
T3	1E-6*	6E-6*	0.199	0.614	1

(c) Mann-Whitney U-test for difference in the average of P_{DD}

	C1	C2	T1	T2	T3
C1	1	< 1E-6*	< 1E-6*	< 1E-6*	1.1E-4*
C2	< 1E-6*	1	< 1E-6*	< 1E-6*	< 1E-6*
T	< 1E-6*	< 1E-6*			
T1	< 1E-6*	< 1E-6*	1	0.061	1E-6*
T2	< 1E-6*	< 1E-6*	0.061	1	< 1E-6*
T3	1.1E-4*	< 1E-6*	1E-6*	< 1E-6*	1

1.3. Individual's response to his/her opponent's behavior in treatment T

To show how a player responds to his/her opponent's behavior (C or D), individual responses (i.e. continue the interaction with the current opponent in the next round, or stop the interaction with the current opponent) to C and D in T1, in T2 and in T3

are shown in Table S5a. Here, the response to C (D) is measured by the probability that the interaction will be kept, or will be stopped. It is easy to see that individuals' responses to C are very similar between T1, T2 and T3 (Table S6). The responses to D are also similar between T1 and T2, but are different between T1 and T3, and between T2 and T3 (Table S6).

Furthermore, we look into how a player using strategy A responds if his\her opponent displays strategy B, where A, B = C, D. The probabilities that a player using strategy A chooses to keep, or stop, the interaction with his\her opponent using strategy B (where A, B = C, D) in the treatments (T1, T2, and T3) are shown in Table S5b. It is easy to see that in all the treatment groups, the probability of a player to choose keep is much higher if the opponent displays C rather than D. In Table S5c we show the probability that players in a particular interaction pair choose to stop or keep, and if the interaction is continued, what the choice combination in the next round is going to be. For the C-C pair, only 10% of the interactions are terminated by choice, and of all the pairs continue to the next round, 88% display C-C, 10% C-D, and only 2% D-D. For C-D pairs, the interactions are stopped by choice at a probability of 56%; and in the next round, 25% of the continued pairs display C-C, 23% C-D, 19% D-C, and 33% D-D. The probability that a D-D interaction pair is stopped by choice is 67%; in the next round, 61% of the interaction pairs stay at D-D, only 6% turn into C-C, and 32% to C-D.

TableS5a. The probabilities that a player chooses to keep, or break, the interaction with his\her opponent using strategy A at the end of each round, where A = C, D.

Group	Choice of a player when his\her opponent uses C		Choice of a player when his\her opponent uses D	
	Keep	Break	Keep	Break
T1	0.91	0.09	0.56	0.44
T2	0.92	0.08	0.56	0.44
T3	0.92	0.08	0.45	0.55
T	0.92	0.08	0.53	0.47

Table S5b. The probabilities that a player using strategy A chooses to keep, or break,

the interaction with his\her opponent using strategy B at the end of each round, where A, B = C, D.

Group	Choice of a player using C in C-C		Choice of a player using C in C-D		Choice of a player using D in D-C		Choice of a player using D in D-D	
	Keep	Break	Keep	Break	Keep	Break	Keep	Break
T1	0.96	0.04	0.76	0.24	0.59	0.41	0.55	0.45
T2	0.94	0.06	0.62	0.38	0.72	0.28	0.60	0.40
T3	0.92	0.08	0.62	0.38	0.62	0.38	0.54	0.46
T	0.94	0.06	0.67	0.33	0.65	0.35	0.57	0.43

Table S5c. The probability that an interaction pair C-C (or C-D, D-D) is broken at the end of each round, and the probability that two players display a particular strategy-pair in the next round if the interaction between these two players is kept.

<table>
<tr><th rowspan="2">Interaction pair</th><th rowspan="2">Probability that an interaction pair is stopped</th><th colspan="4">Probabilities that two subjects display interaction pairs C-C, C-D and D-D in the next round if the interaction between these two subjects is kept</th></tr>
<tr><th>C-C</th><th>C-D</th><th>D-C</th><th>D-D</th></tr>
<tr><td>C-C</td><td>0.13</td><td>0.88</td><td colspan="2">0.05</td><td>0.02</td></tr>
<tr><td>C-D</td><td>0.68</td><td>0.25</td><td>0.23</td><td>0.19</td><td>0.33</td></tr>
<tr><td>D-D</td><td>0.77</td><td>0.06</td><td colspan="2">0.16</td><td>0.61</td></tr>
</table>

Table S6. Mann-Whitney U-test for difference in the average frequencies that a player chooses to keep the interaction after his/her opponent uses strategy A in that round ($n_1 = 60$, $n_2 = 60$) between T1 and T2, between T2 and T3, and between T1 and T3, where A=(C, D). The symbol "*" denotes that the difference is significant at $\alpha = 0.01/3 = 3.3\text{E-}3$ (with Bonferroni correction).

	Test for difference in the average frequencies that a player chooses to keep the interaction after his/her opponent uses C			Test for difference in the average frequencies that a player chooses to keep the interaction after his/her opponent uses D		
	T1	T2	T3	T1	T2	T3
T1	1	0.197	0.262	1	0.902	< 0.001*
T2	0.197	1	0.698	0.902	1	< 0.001*
T3	0.262	0.698	1	< 0.001*	< 0.001*	1

2. Theoretical Analysis

2.1. Evolutionary dynamics for the PD game with payoff matrix $\begin{pmatrix} b-c & -c \\ b & 0 \end{pmatrix}$ where all individuals use OFT

Let P_{CC}, P_{CD} and P_{DD} denote the proportions of interaction pairs C-C, C-D and D-D, respectively. Then, the frequency of C, denoted by x, is given by $x = P_{CC} + P_{CD}/2$, and the frequency of D is $1-x = P_{DD} + P_{CD}/2$. For a large population, the changes in proportions P_{CC}, P_{CD} and P_{DD} between rounds should be considered as fast variables comparing to the change of x since the meeting between a pair of individuals is random. To show this, consider a large population with size N, in which the expected change from x to $x \pm 1/N$ in the time interval $\left(t,\ t+1/N\right)$, denoted by $\Delta x = x(t+1/N) - x(t)$, is

$$\begin{aligned} \langle \Delta x \rangle &\equiv \left\langle x\left(t+1/N\right) - x(t) \right\rangle \\ &= \Pr\left\{\Delta x = 1/N\right\}\left(x(t)+1/N\right) + \Pr\left\{\Delta x = -1/N\right\}\left(x(t)-1/N\right) \\ &\quad + \left[1 - \Pr\left\{\Delta x = 1/N\right\} - \Pr\left\{\Delta x = -1/N\right\}\right] x(t) - x(t) \\ &= \frac{1}{N}\left[\Pr\left\{\Delta x = 1/N\right\} - \Pr\left\{\Delta x = -1/N\right\}\right] \end{aligned}, \qquad \text{[S1]}$$

Where $\Pr\left\{\Delta x = \pm 1/N\right\}$ denotes the probability that Δx equals exactly $\pm 1/N$.

Similarly, the expected changes to P_{CC}, P_{CD} and P_{DD} are given by

$$\begin{aligned} \langle \Delta P_{CC} \rangle &\approx (1-\rho)P_{CC} + \frac{\left(2\rho P_{CC} + P_{CD}\right)^2}{4\left(1-(1-\rho)P_{CC}\right)} - P_{CC}, \\ \langle \Delta P_{CD} \rangle &\approx \frac{\left(2\rho P_{CC} + P_{CD}\right)\left(P_{CD} + 2P_{DD}\right)}{2\left(1-(1-\rho)P_{CC}\right)} - P_{CD}, \\ \langle \Delta P_{DD} \rangle &\approx \frac{\left(P_{CD} + 2P_{DD}\right)^2}{4\left(1-(1-\rho)P_{CC}\right)} - P_{DD}, \end{aligned} \qquad \text{[S2]}$$

respectively. Notice that $\langle \Delta P_{CC} \rangle$, $\langle \Delta P_{CD} \rangle$ and $\langle \Delta P_{DD} \rangle$ are independent of

population size N, and that $\lim_{N\to\infty}\langle\Delta x\rangle = 0$. Thus, for large N, the changes of P_{CC}, P_{CD} and P_{DD} are fast variables comparing to the change of x. Thus, in analogy to the Hardy-Weinberg equilibrium in population genetics (see Ref. 27 in the main text), we can assume that at any time t the proportions P_{CC}, P_{CD} and P_{DD} are at a "temporal equilibrium" for the current value of x, satisfying $P_{CD}^2 = 4\rho P_{CC}P_{DD}$ (SI, Section 2.1). Therefore, we obtain

$$P_{CD} = -\frac{\rho}{1-\rho} + \sqrt{\left(\frac{\rho}{1-\rho}\right)^2 + \frac{4x(1-x)\rho}{1-\rho}} \qquad \text{[S3]}$$

for all $0<x<1$ and $0<\rho<1$ since $P_{CC}+P_{CD}+P_{DD}=1$ and $x = P_{CC}+P_{CD}/2$ (see Figure 3 in the main text).

Notice that, at any time t, an individual using C has an opponent displaying C (respectively, D) with probability $2P_{CC}/\left(2P_{CC}+P_{CD}\right)$ (respectively, $P_{CD}/\left(2P_{CC}+P_{CD}\right)$). Similarly, an individual using D has an opponent displaying C (respectively, D) with probability $P_{CD}/\left(P_{CD}+2P_{DD}\right)$ (respectively, $2P_{DD}/\left(P_{CD}+2P_{DD}\right)$). The expected payoffs of C and D, denoted by π_C and π_D, respectively, are then given by

$$\begin{aligned} \pi_C &= \frac{2P_{CC}}{2P_{CC}+P_{CD}}(b-c) - \frac{P_{CD}}{2P_{CC}+P_{CD}}c = \frac{2x-P_{CD}}{2x}b - c \;, \\ \pi_D &= \frac{P_{CD}}{P_{CD}+2P_{DD}}b = \frac{P_{CD}}{2(1-x)}b \;. \end{aligned} \qquad \text{[S4]}$$

Thus, the time evolution of x can be given by $dx/dt = x(1-x)\left(\pi_C-\pi_D\right)$, i.e., Eq. [2] in the main text.

The boundary $x=0$ of Eq. (2) in the main text is at least locally asymptotically stable since $d\left(dx/dt\right)/dx\,|_{x=0} = -c$, but the boundary $x=1$ must be unstable since $d\left(dx/dt\right)/dx\,|_{x=1} = c$. On the other hand, an interior equilibrium of Eq. [2] in the main text must satisfy $\pi_C-\pi_D=0$, i.e., $x(1-x) = \left(bc/(b-c)^2\right)\left(\rho/(1-\rho)\right)$. Thus, two

interior equilibria, denoted by x_1^* and x_2^*, respectively, with $0 < x_2^* < x_1^* < 1$, exist if $\rho < (b-c)^2/(b+c)^2$, in which case

$$x_{1,2}^* = \frac{1}{2} \pm \sqrt{\frac{1}{4} - \frac{bc}{(b-c)^2} \cdot \frac{\rho}{1-\rho}} \quad ; \qquad \text{[S5]}$$

$x_1^* = x_2^* = 1/2$ if $\rho = (b-c)^2/(b+c)^2$; and no interior equilibrium can exist if $\rho > (b-c)^2/(b+c)^2$. For $\rho = (b-c)^2/(b+c)^2$, the unique interior equilibrium $x^* = 1/2$ must be unstable since $dx/dt < 0$ for all $x \in (0,1)$ except for $x = 1/2$. On the other hand, for $\rho < (b-c)^2/(b+c)^2$, the interior equilibrium $x_1^* > 1/2$ is locally asymptotically stable but x_2^* is unstable since $d\left(dx/dt\right)/dx\,|_{x=x_1^*} < 0$ and $d\left(dx/dt\right)/dx\,|_{x=x_2^*} > 0$.

2.2. Analysis of PD game with payoff matrix $\begin{pmatrix} R & S \\ T & P \end{pmatrix}$ where all players use OFT

For the payoff matrix $\begin{pmatrix} R & S \\ T & P \end{pmatrix}$ with $T > R > P > S$, similar to the analysis in in subsection 2.1, the expected payoffs π_C and π_D are given by

$$\begin{aligned} \pi_C &= \frac{2P_{CC}}{2P_{CC}+P_{CD}}R + \frac{P_{CD}}{2P_{CC}+P_{CD}}S = \frac{2x-P_{CD}}{2x}R + \frac{P_{CD}}{2x}S \ , \\ \pi_D &= \frac{P_{CD}}{P_{CD}+2P_{DD}}T + \frac{2P_{DD}}{P_{CD}+2P_{DD}}P = \frac{P_{CD}}{2(1-x)}T + \frac{2(1-x)-P_{CD}}{2(1-x)}P \ . \end{aligned} \qquad \text{[S6]}$$

Thus, the time evolution of x can be described by

$$\frac{dx}{dt} = x(1-x)(R-P) - \frac{P_{CD}}{2}\left[(1-x)(R-S) + x(T-P)\right] \ , \qquad \text{[S7]}$$

where P_{CD} is at the temporal equilibrium, i.e.

$P_{CD} = -\frac{\rho}{1-\rho} + \sqrt{\left(\frac{\rho}{1-\rho}\right)^2 + \frac{4x(1-x)\rho}{1-\rho}}$ for all $0 < x < 1$. The boundary $x = 1$ is unstable but the boundary $x = 0$ is at least locally asymptotically stable since

$d\left(dx/dt\right)/dx\,|_{x=1}=T-R>0$ and $d\left(dx/dt\right)/dx\,|_{x=0}=S-P<0$. The interior equilibrium of Eq. [S4] is the solution of equation

$$
\begin{aligned}
& x(1-x)(R-P)-\frac{P_{CD}}{2}\left[(1-x)(R-S)+x(T-P)\right]=0 \\
& \Rightarrow\; -\frac{\rho}{1-\rho}+\sqrt{\left(\frac{\rho}{1-\rho}\right)^2+4x(1-x)\frac{\rho}{1-\rho}}=\frac{2x(1-x)(R-P)}{(1-x)(R-S)+x(T-P)}, \qquad \text{[S8]} \\
& \Rightarrow\; x^2-x\left[1-\alpha A(T-S)\right]+\alpha(P-S)(R-S)=0\ ,
\end{aligned}
$$

where

$$
\begin{aligned}
A &= R-S-T+P\ , \\
\alpha &= \frac{\rho/(1-\rho)}{(R-P)^2+\left(\rho/(1-\rho)\right)A^2}\ . \qquad \text{[S9]}
\end{aligned}
$$

So, two possible interior equilibria can be given by

$$
x_{1,2}^{*}=\frac{1+\alpha A(T-S)\pm\sqrt{\left(1+\alpha A(T-S)\right)^2-4\alpha(P-S)(R-S)}}{2}\ . \qquad \text{[S10]}
$$

Here $x_1^*=x_2^*=\left(1+\alpha A(T-S)\right)/2$ if $\left(1+\alpha A(T-S)\right)^2-4\alpha(P-S)(R-S)=0$.

For the local stability of interior equilibrium, a straight forward calculation shows that (i) if only one interior equilibrium exists, then it must be unstable; and (ii) for the situation with two interior equilibria x_1^* and x_2^* (with $x_1^*>x_2^*$), x_1^* is locally asymptotically stable but x_2^* is unstable.
